# Retraction: Exosomal miRNA-34 from cancer-associated fibroblasts inhibits growth and invasion of gastric cancer cells *in vitro* and *in vivo*

**DOI:** 10.18632/aging.205020

**Published:** 2023-08-28

**Authors:** Liang Shi, Zhenyong Wang, Xiuchao Geng, Yuhao Zhang, Ziqing Xue

**Affiliations:** 1 Endoscope Room, Department of General Surgery, Cangzhou Central Hospital, Cangzhou 061001, Hebei Province, China; 2 Medical College of Hebei University, Shijiazhuang 050011, Hebei Province, China; 3 The First Department of General Surgery, Cangzhou Central Hospital, Cangzhou 061001, Hebei Province, China; 4 Integrated Chinese and Western Medicine, Hebei University of Chinese Medicine, Shijiazhuang 050020, Hebei Province, China; 5 Department of Neurosurgery, Affiliated Hospital of Hebei University, Baoding 071000, Hebei Province, China; 6 Hebei University, Baoding 071002, Hebei Province, China

**Keywords:** gastric cancer, cancer-associated fibroblasts, exosome, miRNA-34, proliferation

**This article has been retracted:** Aging has completed the investigation of this paper. We confirmed that **Figure 1A** "Characteristics of control and GC fibroblasts. (**A**) The morphology of fibroblasts from healthy control gastric tissues (CFs) and gastric cancer (GCFs) tissues" and **Figure 7A** and **C** "Exosomal miRNA-34 inhibits tumor growth of GC cells *in vivo*. (**A**) Exosomes derived from GCFs labeled with Cre could be taken up by GC cells of tumor tissue, …(**C**) Representative image of excised xenograft tumors" have internal duplications of the images of the cells and tumors. In addition, three tumor images from **Figure 7C** were later published in the unrelated paper [1]. We also found that transwell assay images in **Figure 3E, H** “Overexpression of miRNA-34 inhibits the proliferation and invasion of GC cell lines. (**E–H**) The invasion of GC cell lines transfected with miRNA-34 mimics” were present in a 2020 article [2] by a different group of authors from a different institution. All authors of the retracted article were notified and all agree to the retraction and signed the retraction letter. The Administration of Cangzhou Central Hospital was also notified and after an institutional investigation, they too recommended retraction of this article.
